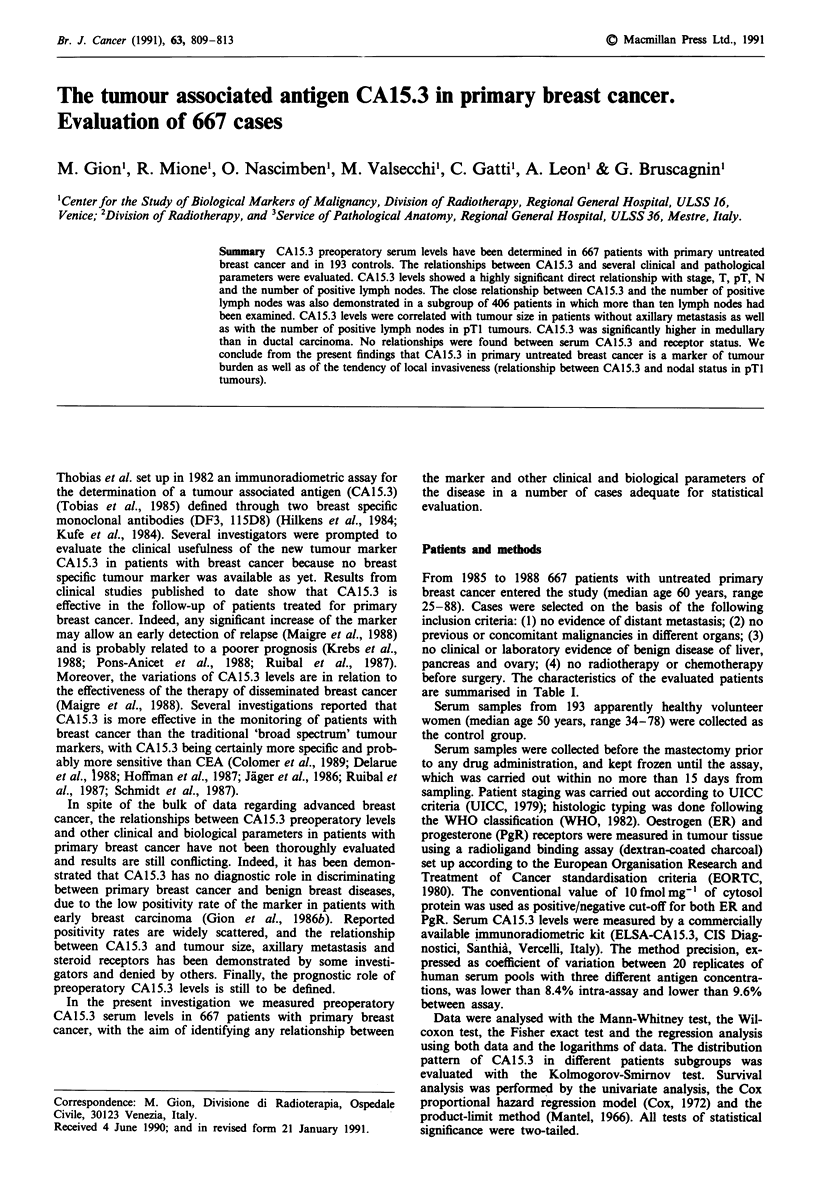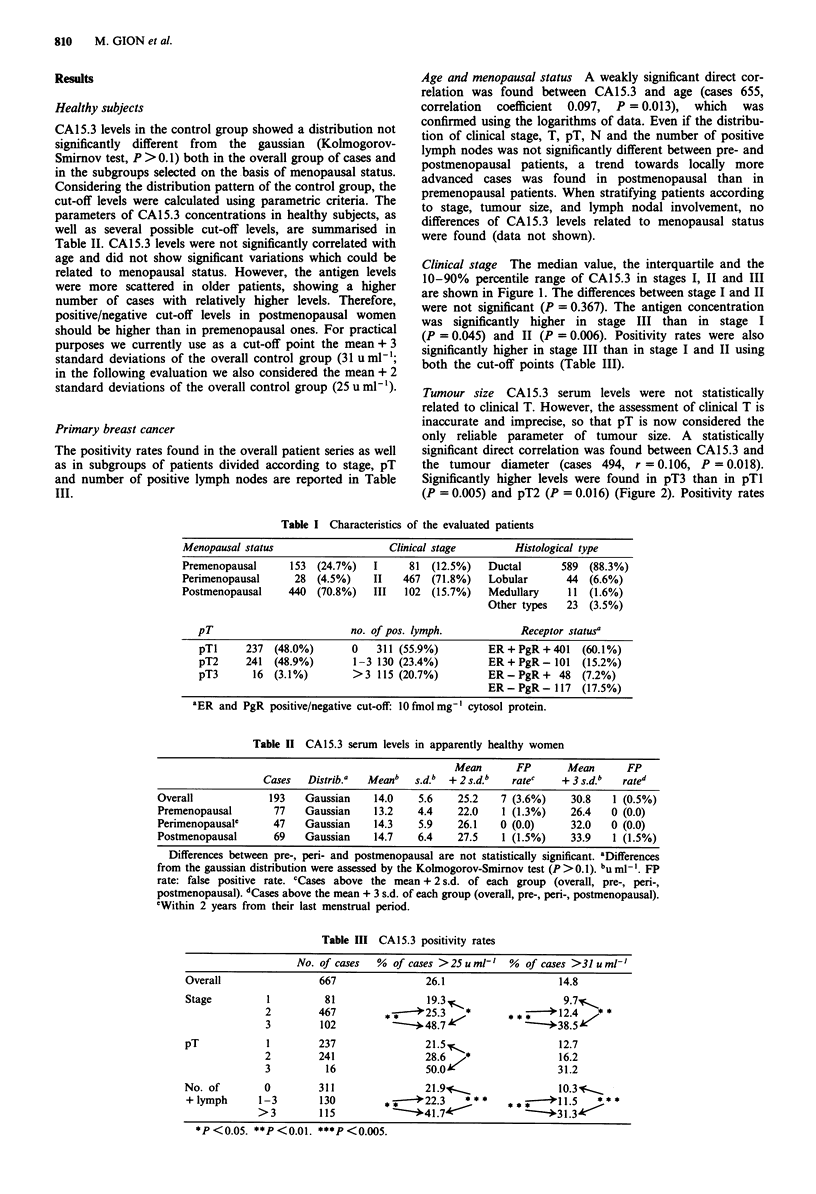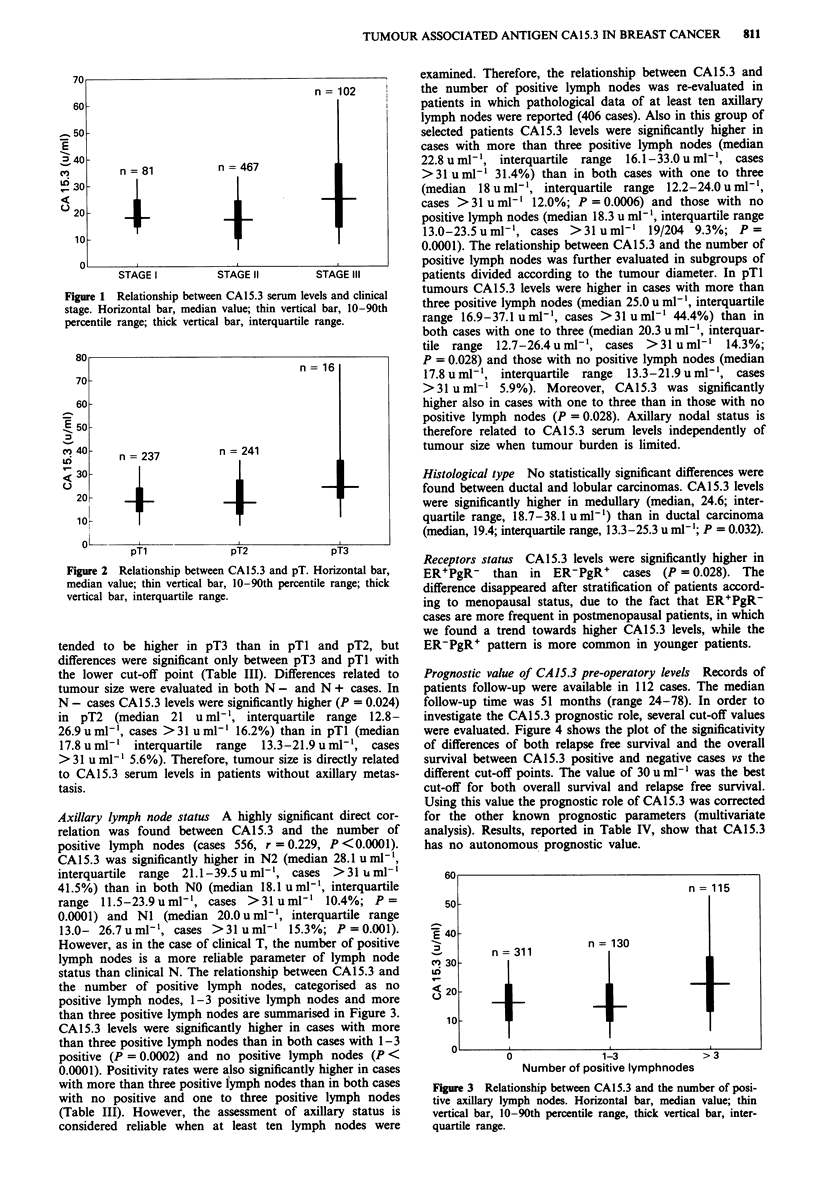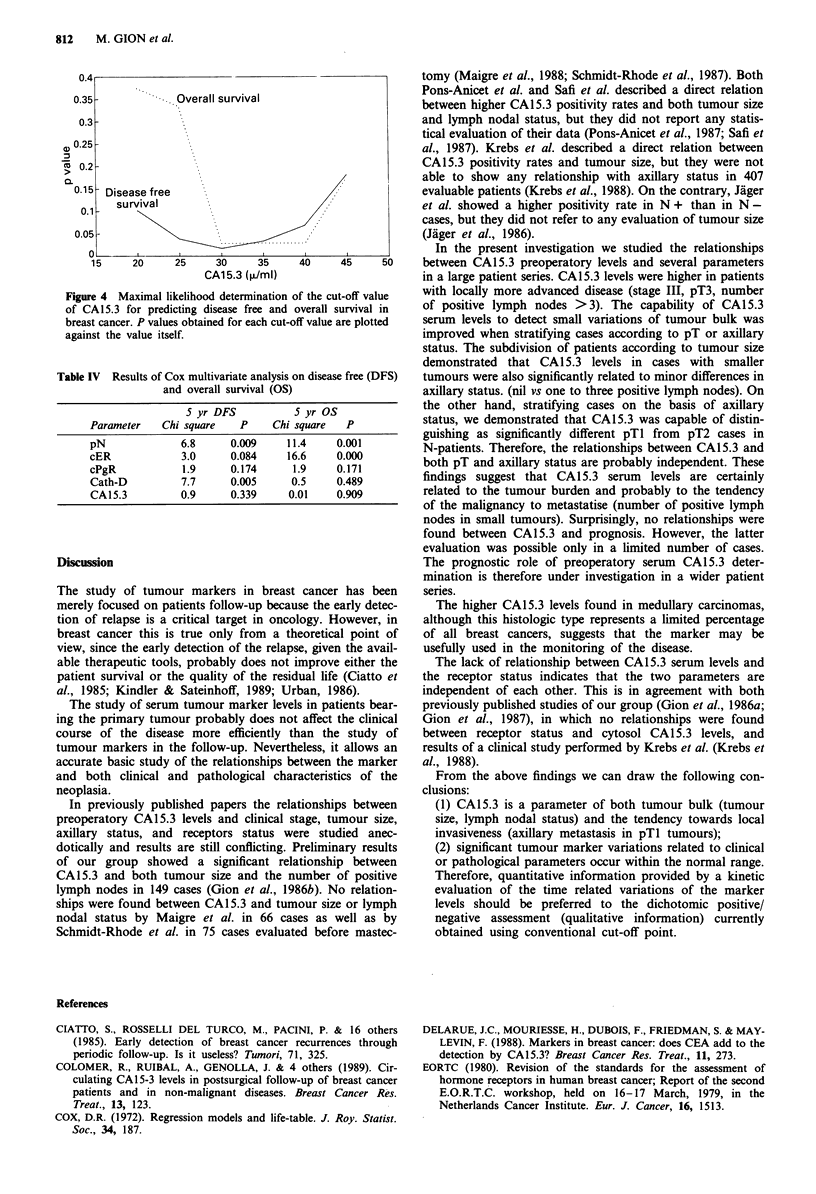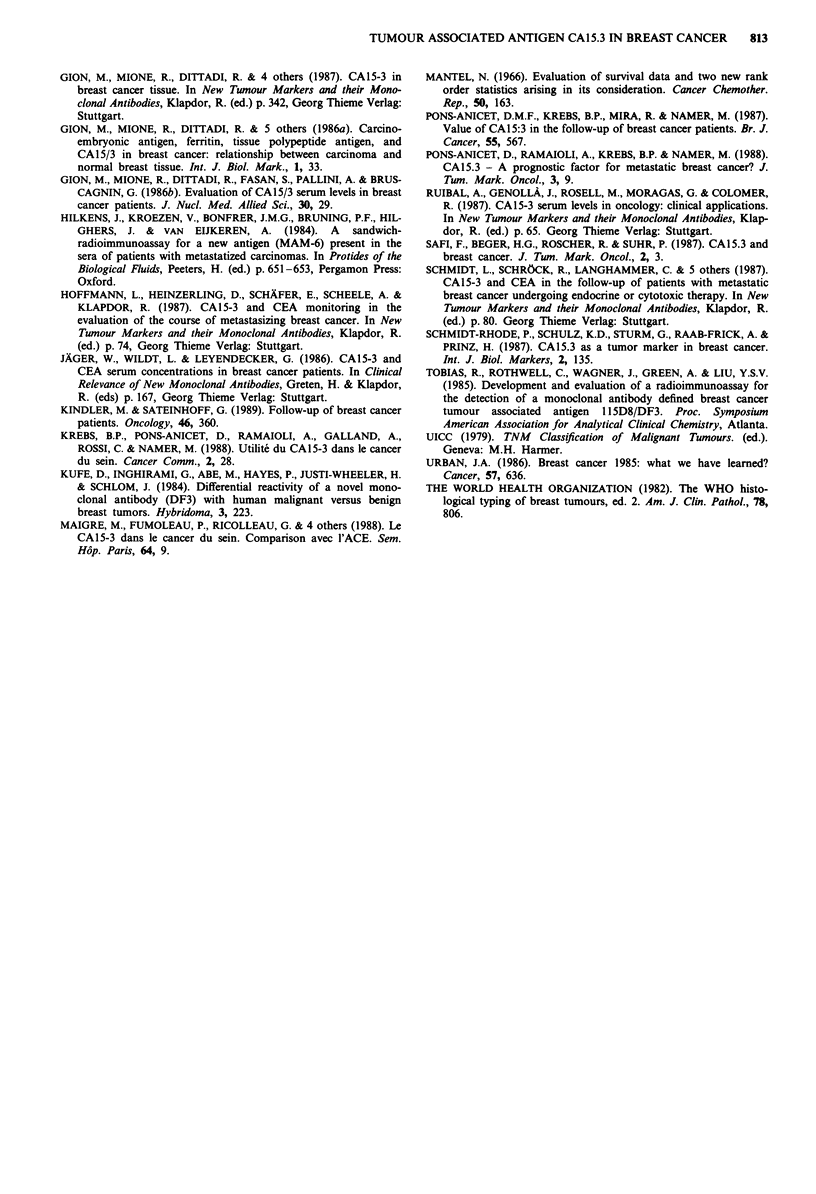# The tumour associated antigen CA15.3 in primary breast cancer. Evaluation of 667 cases.

**DOI:** 10.1038/bjc.1991.179

**Published:** 1991-05

**Authors:** M. Gion, R. Mione, O. Nascimben, M. Valsecchi, C. Gatti, A. Leon, G. Bruscagnin

**Affiliations:** Division of Radiotherapy, Regional General Hospital, ULSS 16, Venice, Italy.

## Abstract

CA15.3 preoperatory serum levels have been determined in 667 patients with primary untreated breast cancer and in 193 controls. The relationships between CA15.3 and several clinical and pathological parameters were evaluated. CA15.3 levels showed a highly significant direct relationship with stage, T, pT, N and the number of positive lymph nodes. The close relationship between CA15.3 and the number of positive lymph nodes was also demonstrated in a subgroup of 406 patients in which more than ten lymph nodes had been examined. CA15.3 levels were correlated with tumour size in patients without axillary metastasis as well as with the number of positive lymph nodes in pT1 tumours. CA15.3 was significantly higher in medullary than in ductal carcinoma. No relationships were found between serum CA15.3 and receptor status. We conclude from the present findings that CA15.3 in primary untreated breast cancer is a marker of tumour burden as well as of the tendency of local invasiveness (relationship between CA15.3 and nodal status in pT1 tumours).


*Br. J. Cancer* (1991), **63**, 809-813 © Macmillan Press Ltd., 1991

### The tumour associated antigen CA15.3 in primary breast cancer. Evaluation of 667 cases

M. Gion[1], R. Mione[1], O. Nascimben[1], M. Valsecchi[1], C. Gatti[1], A. Leon[1] & G. Bruscagnin[1]

[1]*Center for the Study of Biological Markers of Malignancy, Division of Radiotherapy, Regional General Hospital, ULSS 16, Venice;* [2]*Division of Radiotherapy, and* [3]*Service of Pathological Anatomy, Regional General Hospital, ULSS 36, Mestre, Italy.*

**Summary** CA15.3 preoperatory serum levels have been determined in 667 patients with primary untreated breast cancer and in 193 controls. The relationships between CA15.3 and several clinical and pathological parameters were evaluated. CA15.3 levels showed a highly significant direct relationship with stage, T, pT, N and the number of positive lymph nodes. The close relationship between CA15.3 and the number of positive lymph nodes was also demonstrated in a subgroup of 406 patients in which more than ten lymph nodes had been examined. CA15.3 levels were correlated with tumour size in patients without axillary metastasis as well as with the number of positive lymph nodes in pT1 tumours. CA15.3 was significantly higher in medullary than in ductal carcinoma. No relationships were found between serum CA15.3 and receptor status. We conclude from the present findings that CA15.3 in primary untreated breast cancer is a marker of tumour burden as well as of the tendency of local invasiveness (relationship between CA15.3 and nodal status in pT1 tumours).

Thobias *et al.* set up in 1982 an immunoradiometric assay for the determination of a tumour associated antigen (CA15.3) (Tobias *et al.*, 1985) defined through two breast specific monoclonal antibodies (DF3, 115D8) (Hilkens *et al.*, 1984; Kufe *et al.*, 1984). Several investigators were prompted to evaluate the clinical usefulness of the new tumour marker CA15.3 in patients with breast cancer because no breast specific tumour marker was available as yet. Results from clinical studies published to date show that CA15.3 is effective in the follow-up of patients treated for primary breast cancer. Indeed, any significant increase of the marker may allow an early detection of relapse (Maigre *et al.*, 1988) and is probably related to a poorer prognosis (Krebs *et al.*, 1988; Pons-Anicet *et al.*, 1988; Ruibal *et al.*, 1987). Moreover, the variations of CA15.3 levels are in relation to the effectiveness of the therapy of disseminated breast cancer (Maigre *et al.*, 1988). Several investigations reported that CA15.3 is more effective in the monitoring of patients with breast cancer than the traditional 'broad spectrum' tumour markers, with CA15.3 being certainly more specific and probably more sensitive than CEA (Colomer *et al.*, 1989; Delarue *et al.*, 1988; Hoffman *et al.*, 1987; Jäger *et al.*, 1986; Ruibal *et al.*, 1987; Schmidt *et al.*, 1987).

In spite of the bulk of data regarding advanced breast cancer, the relationships between CA15.3 preoperatory levels and other clinical and biological parameters in patients with primary breast cancer have not been thoroughly evaluated and results are still conflicting. Indeed, it has been demonstrated that CA15.3 has no diagnostic role in discriminating between primary breast cancer and benign breast diseases, due to the low positivity rate of the marker in patients with early breast carcinoma (Gion *et al.*, 1986*b*). Reported positivity rates are widely scattered, and the relationship between CA15.3 and tumour size, axillary metastasis and steroid receptors has been demonstrated by some investigators and denied by others. Finally, the prognostic role of preoperatory CA15.3 levels is still to be defined.

In the present investigation we measured preoperatory CA15.3 serum levels in 667 patients with primary breast cancer, with the aim of identifying any relationship between the marker and other clinical and biological parameters of the disease in a number of cases adequate for statistical evaluation.

Correspondence: M. Gion, Divisione di Radioterapia, Ospedale Civile, 30123 Venezia, Italy.
Received 4 June 1990; and in revised form 21 January 1991.

### Patients and methods

From 1985 to 1988 667 patients with untreated primary breast cancer entered the study (median age 60 years, range 25-88). Cases were selected on the basis of the following inclusion criteria: (1) no evidence of distant metastasis; (2) no previous or concomitant malignancies in different organs; (3) no clinical or laboratory evidence of benign disease of liver, pancreas and ovary; (4) no radiotherapy or chemotherapy before surgery. The characteristics of the evaluated patients are summarised in Table I.

Serum samples from 193 apparently healthy volunteer women (median age 50 years, range 34-78) were collected as the control group.

Serum samples were collected before the mastectomy prior to any drug administration, and kept frozen until the assay, which was carried out within no more than 15 days from sampling. Patient staging was carried out according to UICC criteria (UICC, 1979); histologic typing was done following the WHO classification (WHO, 1982). Oestrogen (ER) and progesterone (PgR) receptors were measured in tumour tissue using a radioligand binding assay (dextran-coated charcoal) set up according to the European Organisation Research and Treatment of Cancer standardisation criteria (EORTC, 1980). The conventional value of 10 $fmol\ mg^{-1}$ of cytosol protein was used as positive/negative cut-off for both ER and PgR. Serum CA15.3 levels were measured by a commercially available immunoradiometric kit (ELSA-CA15.3, CIS Diagnostici, Santhià, Vercelli, Italy). The method precision, expressed as coefficient of variation between 20 replicates of human serum pools with three different antigen concentrations, was lower than 8.4% intra-assay and lower than 9.6% between assay.

Data were analysed with the Mann-Whitney test, the Wilcoxon test, the Fisher exact test and the regression analysis using both data and the logarithms of data. The distribution pattern of CA15.3 in different patients subgroups was evaluated with the Kolmogorov-Smirnov test. Survival analysis was performed by the univariate analysis, the Cox proportional hazard regression model (Cox, 1972) and the product-limit method (Mantel, 1966). All tests of statistical significance were two-tailed.

### Results

### Healthy subjects

CA15.3 levels in the control group showed a distribution not significantly different from the gaussian (Kolmogorov-Smirnov test, $P > 0.1$) both in the overall group of cases and in the subgroups selected on the basis of menopausal status. Considering the distribution pattern of the control group, the cut-off levels were calculated using parametric criteria. The parameters of CA15.3 concentrations in healthy subjects, as well as several possible cut-off levels, are summarised in Table II. CA15.3 levels were not significantly correlated with age and did not show significant variations which could be related to menopausal status. However, the antigen levels were more scattered in older patients, showing a higher number of cases with relatively higher levels. Therefore, positive/negative cut-off levels in postmenopausal women should be higher than in premenopausal ones. For practical purposes we currently use as a cut-off point the mean + 3 standard deviations of the overall control group (31 u $ml^{-1}$; in the following evaluation we also considered the mean + 2 standard deviations of the overall control group (25 u $ml^{-1}$).

### Primary breast cancer

The positivity rates found in the overall patient series as well as in subgroups of patients divided according to stage, pT and number of positive lymph nodes are reported in Table III.

*Age and menopausal status* A weakly significant direct correlation was found between CA15.3 and age (cases 655, correlation coefficient 0.097, $P = 0.013$), which was confirmed using the logarithms of data. Even if the distribution of clinical stage, T, pT, N and the number of positive lymph nodes was not significantly different between pre- and postmenopausal patients, a trend towards locally more advanced cases was found in postmenopausal than in premenopausal patients. When stratifying patients according to stage, tumour size, and lymph nodal involvement, no differences of CA15.3 levels related to menopausal status were found (data not shown).

*Clinical stage* The median value, the interquartile and the 10–90% percentile range of CA15.3 in stages I, II and III are shown in Figure 1. The differences between stage I and II were not significant ($P = 0.367$). The antigen concentration was significantly higher in stage III than in stage I ($P = 0.045$) and II ($P = 0.006$). Positivity rates were also significantly higher in stage III than in stage I and II using both the cut-off points (Table III).

*Tumour size* CA15.3 serum levels were not statistically related to clinical T. However, the assessment of clinical T is inaccurate and imprecise, so that pT is now considered the only reliable parameter of tumour size. A statistically significant direct correlation was found between CA15.3 and the tumour diameter (cases 494, $r = 0.106$, $P = 0.018$). Significantly higher levels were found in pT3 than in pT1 ($P = 0.005$) and pT2 ($P = 0.016$) (Figure 2). Positivity rates

**Table I** Characteristics of the evaluated patients

| *Menopausal status* | | | *Clinical stage* | | | *Histological type* | | |
|---|---|---|---|---|---|---|---|---|
| Premenopausal | 153 | (24.7%) | I | 81 | (12.5%) | Ductal | 589 | (88.3%) |
| Perimenopausal | 28 | (4.5%) | II | 467 | (71.8%) | Lobular | 44 | (6.6%) |
| Postmenopausal | 440 | (70.8%) | III | 102 | (15.7%) | Medullary | 11 | (1.6%) |
| | | | | | | Other types | 23 | (3.5%) |

| *pT* | | | *no. of pos. lymph.* | | | *Receptor status*[a] | | |
|---|---|---|---|---|---|---|---|---|
| pT1 | 237 | (48.0%) | 0 | 311 | (55.9%) | ER + PgR + | 401 | (60.1%) |
| pT2 | 241 | (48.9%) | 1–3 | 130 | (23.4%) | ER + PgR – | 101 | (15.2%) |
| pT3 | 16 | (3.1%) | >3 | 115 | (20.7%) | ER – PgR + | 48 | (7.2%) |
| | | | | | | ER – PgR – | 117 | (17.5%) |

[a]ER and PgR positive/negative cut-off: 10 fmol $mg^{-1}$ cytosol protein.

**Table II** CA15.3 serum levels in apparently healthy women

| | *Cases* | *Distrib.*[a] | *Mean*[b] | *s.d.*[b] | *Mean + 2 s.d.*[b] | *FP rate*[c] | *Mean + 3 s.d.*[b] | *FP rate*[d] |
|---|---|---|---|---|---|---|---|---|
| Overall | 193 | Gaussian | 14.0 | 5.6 | 25.2 | 7 (3.6%) | 30.8 | 1 (0.5%) |
| Premenopausal | 77 | Gaussian | 13.2 | 4.4 | 22.0 | 1 (1.3%) | 26.4 | 0 (0.0) |
| Perimenopausal[e] | 47 | Gaussian | 14.3 | 5.9 | 26.1 | 0 (0.0) | 32.0 | 0 (0.0) |
| Postmenopausal | 69 | Gaussian | 14.7 | 6.4 | 27.5 | 1 (1.5%) | 33.9 | 1 (1.5%) |

Differences between pre-, peri- and postmenopausal are not statistically significant. [a]Differences from the gaussian distribution were assessed by the Kolmogorov-Smirnov test ($P > 0.1$). [b]u $ml^{-1}$. FP rate: false positive rate. [c]Cases above the mean + 2 s.d. of each group (overall, pre-, peri-, postmenopausal). [d]Cases above the mean + 3 s.d. of each group (overall, pre-, peri-, postmenopausal). [e]Within 2 years from their last menstrual period.

**Table III** CA15.3 positivity rates

| | | *No. of cases* | *% of cases >25 u ml⁻¹* | *% of cases >31 u ml⁻¹* |
|---|---|---|---|---|
| Overall | | 667 | 26.1 | 14.8 |
| Stage | 1 | 81 | 19.3 | 9.7 |
| | 2 | 467 | ** 25.3 * | *** 12.4 ** |
| | 3 | 102 | 48.7 | 38.5 |
| pT | 1 | 237 | 21.5 | 12.7 |
| | 2 | 241 | 28.6 * | 16.2 |
| | 3 | 16 | 50.0 | 31.2 |
| No. of + lymph | 0 | 311 | 21.9 | 10.3 |
| | 1–3 | 130 | * 22.3 *** | *** 11.5 *** |
| | >3 | 115 | 41.7 | 31.3 |

*$P < 0.05$. **$P < 0.01$. ***$P < 0.005$.

**Figure 1** Relationship between CA15.3 serum levels and clinical stage. Horizontal bar, median value; thin vertical bar, 10–90th percentile range; thick vertical bar, interquartile range.

**Figure 2** Relationship between CA15.3 and pT. Horizontal bar, median value; thin vertical bar, 10–90th percentile range; thick vertical bar, interquartile range.

tended to be higher in pT3 than in pT1 and pT2, but differences were significant only between pT3 and pT1 with the lower cut-off point (Table III). Differences related to tumour size were evaluated in both N – and N + cases. In N – cases CA15.3 levels were significantly higher ($P = 0.024$) in pT2 (median 21 u $ml^{-1}$, interquartile range 12.8–26.9 u $ml^{-1}$, cases $>$31 u $ml^{-1}$ 16.2%) than in pT1 (median 17.8 u $ml^{-1}$ interquartile range 13.3–21.9 u $ml^{-1}$, cases $>$31 u $ml^{-1}$ 5.6%). Therefore, tumour size is directly related to CA15.3 serum levels in patients without axillary metastasis.

*Axillary lymph node status* A highly significant direct correlation was found between CA15.3 and the number of positive lymph nodes (cases 556, $r = 0.229$, $P < 0.0001$). CA15.3 was significantly higher in N2 (median 28.1 u $ml^{-1}$, interquartile range 21.1–39.5 u $ml^{-1}$, cases $>$31 u $ml^{-1}$ 41.5%) than in both N0 (median 18.1 u $ml^{-1}$, interquartile range 11.5–23.9 u $ml^{-1}$, cases $>$31 u $ml^{-1}$ 10.4%; $P =$ 0.0001) and N1 (median 20.0 u $ml^{-1}$, interquartile range 13.0– 26.7 u $ml^{-1}$, cases $>$31 u $ml^{-1}$ 15.3%; $P = 0.001$). However, as in the case of clinical T, the number of positive lymph nodes is a more reliable parameter of lymph node status than clinical N. The relationship between CA15.3 and the number of positive lymph nodes, categorised as no positive lymph nodes, 1–3 positive lymph nodes and more than three positive lymph nodes are summarised in Figure 3. CA15.3 levels were significantly higher in cases with more than three positive lymph nodes than in both cases with 1–3 positive ($P = 0.0002$) and no positive lymph nodes ($P <$ 0.0001). Positivity rates were also significantly higher in cases with more than three positive lymph nodes than in both cases with no positive and one to three positive lymph nodes (Table III). However, the assessment of axillary status is considered reliable when at least ten lymph nodes were examined. Therefore, the relationship between CA15.3 and the number of positive lymph nodes was re-evaluated in patients in which pathological data of at least ten axillary lymph nodes were reported (406 cases). Also in this group of selected patients CA15.3 levels were significantly higher in cases with more than three positive lymph nodes (median 22.8 u $ml^{-1}$, interquartile range 16.1–33.0 u $ml^{-1}$, cases $>$31 u $ml^{-1}$ 31.4%) than in both cases with one to three (median 18 u $ml^{-1}$, interquartile range 12.2–24.0 u $ml^{-1}$, cases $>$31 u $ml^{-1}$ 12.0%; $P = 0.0006$) and those with no positive lymph nodes (median 18.3 u $ml^{-1}$, interquartile range 13.0–23.5 u $ml^{-1}$, cases $>$31 u $ml^{-1}$ 19/204 9.3%; $P =$ 0.0001). The relationship between CA15.3 and the number of positive lymph nodes was further evaluated in subgroups of patients divided according to the tumour diameter. In pT1 tumours CA15.3 levels were higher in cases with more than three positive lymph nodes (median 25.0 u $ml^{-1}$, interquartile range 16.9–37.1 u $ml^{-1}$, cases $>$31 u $ml^{-1}$ 44.4%) than in both cases with one to three (median 20.3 u $ml^{-1}$, interquartile range 12.7–26.4 u $ml^{-1}$, cases $>$31 u $ml^{-1}$ 14.3%; $P = 0.028$) and those with no positive lymph nodes (median 17.8 u $ml^{-1}$, interquartile range 13.3–21.9 u $ml^{-1}$, cases $>$31 u $ml^{-1}$ 5.9%). Moreover, CA15.3 was significantly higher also in cases with one to three than in those with no positive lymph nodes ($P = 0.028$). Axillary nodal status is therefore related to CA15.3 serum levels independently of tumour size when tumour burden is limited.

*Histological type* No statistically significant differences were found between ductal and lobular carcinomas. CA15.3 levels were significantly higher in medullary (median, 24.6; interquartile range, 18.7–38.1 u $ml^{-1}$) than in ductal carcinoma (median, 19.4; interquartile range, 13.3–25.3 u $ml^{-1}$; $P = 0.032$).

*Receptors status* CA15.3 levels were significantly higher in $ER^+PgR^-$ than in $ER^-PgR^+$ cases ($P = 0.028$). The difference disappeared after stratification of patients according to menopausal status, due to the fact that $ER^+PgR^-$ cases are more frequent in postmenopausal patients, in which we found a trend towards higher CA15.3 levels, while the $ER^-PgR^+$ pattern is more common in younger patients.

*Prognostic value of CA15.3 pre-operatory levels* Records of patients follow-up were available in 112 cases. The median follow-up time was 51 months (range 24–78). In order to investigate the CA15.3 prognostic role, several cut-off values were evaluated. Figure 4 shows the plot of the significativity of differences of both relapse free survival and the overall survival between CA15.3 positive and negative cases *vs* the different cut-off points. The value of 30 u $ml^{-1}$ was the best cut-off for both overall survival and relapse free survival. Using this value the prognostic role of CA15.3 was corrected for the other known prognostic parameters (multivariate analysis). Results, reported in Table IV, show that CA15.3 has no autonomous prognostic value.

**Figure 3** Relationship between CA15.3 and the number of positive axillary lymph nodes. Horizontal bar, median value; thin vertical bar, 10–90th percentile range, thick vertical bar, interquartile range.

**Figure 4** Maximal likelihood determination of the cut-off value of CA15.3 for predicting disease free and overall survival in breast cancer. *P* values obtained for each cut-off value are plotted against the value itself.

**Table IV** Results of Cox multivariate analysis on disease free (DFS) and overall survival (OS)

| | *5 yr DFS* | | *5 yr OS* | |
|---|---|---|---|---|
| *Parameter* | *Chi square* | *P* | *Chi square* | *P* |
| pN | 6.8 | 0.009 | 11.4 | 0.001 |
| cER | 3.0 | 0.084 | 16.6 | 0.000 |
| cPgR | 1.9 | 0.174 | 1.9 | 0.171 |
| Cath-D | 7.7 | 0.005 | 0.5 | 0.489 |
| CA15.3 | 0.9 | 0.339 | 0.01 | 0.909 |

### Discussion

The study of tumour markers in breast cancer has been merely focused on patients follow-up because the early detection of relapse is a critical target in oncology. However, in breast cancer this is true only from a theoretical point of view, since the early detection of the relapse, given the available therapeutic tools, probably does not improve either the patient survival or the quality of the residual life (Ciatto *et al.*, 1985; Kindler & Sateinhoff, 1989; Urban, 1986).

The study of serum tumour marker levels in patients bearing the primary tumour probably does not affect the clinical course of the disease more efficiently than the study of tumour markers in the follow-up. Nevertheless, it allows an accurate basic study of the relationships between the marker and both clinical and pathological characteristics of the neoplasia.

In previously published papers the relationships between preoperatory CA15.3 levels and clinical stage, tumour size, axillary status, and receptors status were studied anecdotically and results are still conflicting. Preliminary results of our group showed a significant relationship between CA15.3 and both tumour size and the number of positive lymph nodes in 149 cases (Gion *et al.*, 1986*b*). No relationships were found between CA15.3 and tumour size or lymph nodal status by Maigre *et al.* in 66 cases as well as by Schmidt-Rhode *et al.* in 75 cases evaluated before mastectomy (Maigre *et al.*, 1988; Schmidt-Rhode *et al.*, 1987). Both Pons-Anicet *et al.* and Safi *et al.* described a direct relation between higher CA15.3 positivity rates and both tumour size and lymph nodal status, but they did not report any statistical evaluation of their data (Pons-Anicet *et al.*, 1987; Safi *et al.*, 1987). Krebs *et al.* described a direct relation between CA15.3 positivity rates and tumour size, but they were not able to show any relationship with axillary status in 407 evaluable patients (Krebs *et al.*, 1988). On the contrary, Jäger *et al.* showed a higher positivity rate in N+ than in N− cases, but they did not refer to any evaluation of tumour size (Jäger *et al.*, 1986).

In the present investigation we studied the relationships between CA15.3 preoperatory levels and several parameters in a large patient series. CA15.3 levels were higher in patients with locally more advanced disease (stage III, pT3, number of positive lymph nodes >3). The capability of CA15.3 serum levels to detect small variations of tumour bulk was improved when stratifying cases according to pT or axillary status. The subdivision of patients according to tumour size demonstrated that CA15.3 levels in cases with smaller tumours were also significantly related to minor differences in axillary status. (nil *vs* one to three positive lymph nodes). On the other hand, stratifying cases on the basis of axillary status, we demonstrated that CA15.3 was capable of distinguishing as significantly different pT1 from pT2 cases in N-patients. Therefore, the relationships between CA15.3 and both pT and axillary status are probably independent. These findings suggest that CA15.3 serum levels are certainly related to the tumour burden and probably to the tendency of the malignancy to metastatise (number of positive lymph nodes in small tumours). Surprisingly, no relationships were found between CA15.3 and prognosis. However, the latter evaluation was possible only in a limited number of cases. The prognostic role of preoperatory serum CA15.3 determination is therefore under investigation in a wider patient series.

The higher CA15.3 levels found in medullary carcinomas, although this histologic type represents a limited percentage of all breast cancers, suggests that the marker may be usefully used in the monitoring of the disease.

The lack of relationship between CA15.3 serum levels and the receptor status indicates that the two parameters are independent of each other. This is in agreement with both previously published studies of our group (Gion *et al.*, 1986*a*; Gion *et al.*, 1987), in which no relationships were found between receptor status and cytosol CA15.3 levels, and results of a clinical study performed by Krebs *et al.* (Krebs *et al.*, 1988).

From the above findings we can draw the following conclusions:

(1) CA15.3 is a parameter of both tumour bulk (tumour size, lymph nodal status) and the tendency towards local invasiveness (axillary metastasis in pT1 tumours);

(2) significant tumour marker variations related to clinical or pathological parameters occur within the normal range. Therefore, quantitative information provided by a kinetic evaluation of the time related variations of the marker levels should be preferred to the dichotomic positive/negative assessment (qualitative information) currently obtained using conventional cut-off point.

### References

CIATTO, S., ROSSELLI DEL TURCO, M., PACINI, P. & 16 others (1985). Early detection of breast cancer recurrences through periodic follow-up. Is it useless? *Tumori*, 71, 325.

COLOMER, R., RUIBAL, A., GENOLLA, J. & 4 others (1989). Circulating CA15-3 levels in postsurgical follow-up of breast cancer patients and in non-malignant diseases. *Breast Cancer Res. Treat.*, **13**, 123.

COX, D.R. (1972). Regression models and life-table. *J. Roy. Statist. Soc.*, **34**, 187.

DELARUE, J.C., MOURIESSE, H., DUBOIS, F., FRIEDMAN, S. & MAY-LEVIN, F. (1988). Markers in breast cancer: does CEA add to the detection by CA15.3? *Breast Cancer Res. Treat.*, **11**, 273.

EORTC (1980). Revision of the standards for the assessment of hormone receptors in human breast cancer; Report of the second E.O.R.T.C. workshop, held on 16–17 March, 1979, in the Netherlands Cancer Institute. *Eur. J. Cancer*, **16**, 1513.

GION, M., MIONE, R., DITTADI, R. & 4 others (1987). CA15-3 in breast cancer tissue. In *New Tumour Markers and their Monoclonal Antibodies*, Klapdor, R. (ed.) p. 342, Georg Thieme Verlag: Stuttgart.

GION, M., MIONE, R., DITTADI, R. & 5 others (1986*a*). Carcinoembryonic antigen, ferritin, tissue polypeptide antigen, and CA15/3 in breast cancer: relationship between carcinoma and normal breast tissue. *Int. J. Biol. Mark.*, **1**, 33.

GION, M., MIONE, R., DITTADI, R., FASAN, S., PALLINI, A. & BRUSCAGNIN, G. (1986*b*). Evaluation of CA15/3 serum levels in breast cancer patients. *J. Nucl. Med. Allied Sci.*, **30**, 29.

HILKENS, J., KROEZEN, V., BONFRER, J.M.G., BRUNING, P.F., HILGHERS, J. & VAN EIJKEREN, A. (1984). A sandwich-radioimmunoassay for a new antigen (MAM-6) present in the sera of patients with metastatized carcinomas. In *Protides of the Biological Fluids*, Peeters, H. (ed.) p. 651–653, Pergamon Press: Oxford.

HOFFMANN, L., HEINZERLING, D., SCHÄFER, E., SCHEELE, A. & KLAPDOR, R. (1987). CA15-3 and CEA monitoring in the evaluation of the course of metastasizing breast cancer. In *New Tumour Markers and their Monoclonal Antibodies*, Klapdor, R. (ed.) p. 74, Georg Thieme Verlag: Stuttgart.

JÄGER, W., WILDT, L. & LEYENDECKER, G. (1986). CA15-3 and CEA serum concentrations in breast cancer patients. In *Clinical Relevance of New Monoclonal Antibodies*, Greten, H. & Klapdor, R. (eds) p. 167, Georg Thieme Verlag: Stuttgart.

KINDLER, M. & SATEINHOFF, G. (1989). Follow-up of breast cancer patients. *Oncology*, **46**, 360.

KREBS, B.P., PONS-ANICET, D., RAMAIOLI, A., GALLAND, A., ROSSI, C. & NAMER, M. (1988). Utilité du CA15-3 dans le cancer du sein. *Cancer Comm.*, **2**, 28.

KUFE, D., INGHIRAMI, G., ABE, M., HAYES, P., JUSTI-WHEELER, H. & SCHLOM, J. (1984). Differential reactivity of a novel monoclonal antibody (DF3) with human malignant versus benign breast tumors. *Hybridoma*, **3**, 223.

MAIGRE, M., FUMOLEAU, P., RICOLLEAU, G. & 4 others (1988). Le CA15-3 dans le cancer du sein. Comparison avec l'ACE. *Sem. Hôp. Paris*, **64**, 9.

MANTEL, N. (1966). Evaluation of survival data and two new rank order statistics arising in its consideration. *Cancer Chemother. Rep.*, **50**, 163.

PONS-ANICET, D.M.F., KREBS, B.P., MIRA, R. & NAMER, M. (1987). Value of CA15:3 in the follow-up of breast cancer patients. *Br. J. Cancer*, **55**, 567.

PONS-ANICET, D., RAMAIOLI, A., KREBS, B.P. & NAMER, M. (1988). CA15.3 – A prognostic factor for metastatic breast cancer? *J. Tum. Mark. Oncol.*, **3**, 9.

RUIBAL, A., GENOLLÂ, J., ROSELL, M., MORAGAS, G. & COLOMER, R. (1987). CA15-3 serum levels in oncology: clinical applications. In *New Tumour Markers and their Monoclonal Antibodies*, Klapdor, R. (ed.) p. 65. Georg Thieme Verlag: Stuttgart.

SAFI, F., BEGER, H.G., ROSCHER, R. & SUHR, P. (1987). CA15.3 and breast cancer. *J. Tum. Mark. Oncol.*, **2**, 3.

SCHMIDT, L., SCHRÖCK, R., LANGHAMMER, C. & 5 others (1987). CA15-3 and CEA in the follow-up of patients with metastatic breast cancer undergoing endocrine or cytotoxic therapy. In *New Tumour Markers and their Monoclonal Antibodies*, Klapdor, R. (ed.) p. 80. Georg Thieme Verlag: Stuttgart.

SCHMIDT-RHODE, P., SCHULZ, K.D., STURM, G., RAAB-FRICK, A. & PRINZ, H. (1987). CA15.3 as a tumor marker in breast cancer. *Int. J. Biol. Markers*, **2**, 135.

TOBIAS, R., ROTHWELL, C., WAGNER, J., GREEN, A. & LIU, Y.S.V. (1985). Development and evaluation of a radioimmunoassay for the detection of a monoclonal antibody defined breast cancer tumour associated antigen 115D8/DF3. *Proc. Symposium American Association for Analytical Clinical Chemistry*, Atlanta.

UICC (1979). *TNM Classification of Malignant Tumours*. (ed.). Geneva: M.H. Harmer.

URBAN, J.A. (1986). Breast cancer 1985: what we have learned? *Cancer*, **57**, 636.

THE WORLD HEALTH ORGANIZATION (1982). The WHO histological typing of breast tumours, ed. 2. *Am. J. Clin. Pathol.*, **78**, 806.